# Satisfaction and Usability of an Information and Communications Technology–Based System by Clinically Healthy Patients With COVID-19 and Medical Professionals: Cross-sectional Survey and Focus Group Interview Study

**DOI:** 10.2196/26227

**Published:** 2021-08-26

**Authors:** Ye Seul Bae, Kyung Hwan Kim, Sae Won Choi, Taehoon Ko, Jun Seo Lim, Meihua Piao

**Affiliations:** 1 Office of Hospital Information Seoul National University Hospital Seoul Republic of Korea; 2 Department of Thoracic & Cardiovascular Surgery Seoul National University Hospital Seoul Republic of Korea; 3 Department of Thoracic & Cardiovascular Surgery Seoul National University College of Medicine Seoul Republic of Korea; 4 Department of Medical Informatics Catholic University of Korea Seoul Republic of Korea

**Keywords:** COVID-19, mobile app, telemedicine, wearable device, vital sign, satisfaction, usability

## Abstract

**Background:**

Digital health care is an important strategy in the war against COVID-19. South Korea introduced living and treatment support centers (LTSCs) to control regional outbreaks and care for patients with asymptomatic or mild COVID-19. Seoul National University Hospital (SNUH) introduced information and communications technology (ICT)–based solutions to manage clinically healthy patients with COVID-19.

**Objective:**

This study aims to investigate satisfaction and usability by patients and health professionals in the optimal use of a mobile app and wearable device that SNUH introduced to the LTSC for clinically healthy patients with COVID-19.

**Methods:**

Online surveys and focus group interviews were conducted to collect quantitative and qualitative data.

**Results:**

Regarding usability testing of the wearable device, perceived usefulness had the highest mean score of 4.45 (SD 0.57) points out of 5. Regarding usability of the mobile app, perceived usefulness had the highest mean score of 4.62 (SD 0.48) points out of 5. Regarding satisfaction items for the mobile app among medical professionals, the “self-reporting” item had the highest mean score of 4.42 (SD 0.58) points out of 5. In focus group interviews of health care professionals, hospital information system interfacing was the most important functional requirement for ICT-based COVID-19 telemedicine.

**Conclusions:**

Improvement of patient safety and reduction of the burden on medical staff were the expected positive outcomes. Stability and reliability of the device, patient education, accountability, and reimbursement issues should be considered as part of the development of remote patient monitoring. In responding to a novel contagious disease, telemedicine and a wearable device were shown to be useful during a global crisis.

## Introduction

The outbreak of COVID-19 has caused major concerns worldwide. On March 11, 2020, the World Health Organization (WHO) designated COVID-19 as a pandemic and it continues to rapidly spread in almost every country across the globe [1]. As of November 29, 2020, up to 61 million confirmed cases and 1.4 million deaths were reported according to the WHO [2]. In late February 2020, many infections occurred in the Daegu-Gyeongbuk region located in the southeastern part of South Korea due to mass spread at religious facilities. Although every patient should be treated in a negative pressure isolation room in order to minimize spread, there were insufficient medical facilities and medical personnel due to the rapid increase in the number of confirmed patients [3,4].

The Korean government recommended that mild or asymptomatic patients with a positive COVID-19 test be admitted to a living and treatment support center (LTSC) and be managed each center [4]. At the government’s request, Seoul National University Hospital (SNUH) operated the third LTSC at the SNUH Human Resource Development Center in Mungyeong, Gyeongsangbuk Province, 180 km southeast of Seoul and 100 km northwest of Daegu, from March 5 to April 9, 2020.

SNUH introduced novel strategies applying information and communications technology (ICT)–based remote patient management systems to a COVID-19 LTSC according to patient clinical pathways [5]. These approaches included cloud-based medical image sharing when a patient was admitted or transferred, communication through mobile apps and wearable monitoring devices for remote consultation, electronic health record templates in hospital information systems (HISs), dashboards for patient monitoring, and an e-prescription system to facilitate management of clinically healthy patients with COVID-19 [5].

Digital health care is an important strategy in the war against COVID-19, as it can minimize the spread of infection and contribute to diagnosis [6-9], treatment [10,11], and management [12,13] after discharge. This study aims to provide insight into the optimal use of the mobile apps and wearable devices that SNUH introduced to the LTSC through surveys and focus group interviews.

## Methods

### Overview

In this study, online surveys and focus group interviews were conducted to collect quantitative and qualitative data exploring experiences of the ICT-based clinical system for the LTSC operated by SNUH. The study was approved by the institutional review board of SNUH (2004-026-1115).

### Participants

#### Patients

For quantitative data collection, an online survey was conducted. All respondents were adult males and females who agreed to participate in the survey. From the time the LTSC opened on March 5, 2020, until it closed on April 9, 2020, a total of 118 patients had been admitted [5]. Since clinically healthy patients with COVID-19 were admitted to the LTSC, there was no definite indication for use of a wearable device according to severity of symptoms. We allocated a wearable vital-sign data recorder (VDR)—the VDR-1000 (TriBell Labs)—to each of the first 10 rooms, which could accommodate 12 patients each. During the 36 days of LTSC operation, 24 patients admitted to the 10 rooms used the VDR-1000. The survey period was from April 20 to 24, 2020. Mobile text messages were sent to all patients asking them to visit a given web link to access the mobile app survey; 24 patients used the wearable device for the continuous monitoring survey with additional instructions. Finally, 12 respondents completed the mobile app usability survey, and 11 completed surveys on continuous remote monitoring. We explained the study details and obtained informed consent from patients who agreed to participate in the study; participants received ₩10,000 (US $8.60) as compensation.

#### Medical Staff

All respondents were medical staff (ie, physicians and nurses) who had worked at the LTSC of SNUH. The survey period was from April 20 to April 24, 2020. An SMS message was sent to participants asking them to visit a given web link with additional instructions, and those who agreed to participate were invited to complete the survey. A total of 24 respondents answered the questionnaire. Participants in the study received ₩10,000 (US $8.60) as compensation.

### Quantitative Data Collection

Two separate online surveys were designed for patients: one regarding the mobile app used for self-reporting, communication, and notifications, and another regarding the wearable device used for remote monitoring. For both surveys, questions about perceived usefulness, perceived ease of use, and satisfaction were included. Perceived usefulness is a subjective belief that the productivity and efficiency of work will be increased by introducing a new technology or system. Perceived ease of use is the subjective belief that using a new system will not require much mental and physical effort. Medical staff were surveyed regarding their satisfaction with the overall ICT system of the LTSC [14-16].

Respondents rated their level of perceived importance of the device and mobile app using a 5-point Likert scale with the following response options and scores: strongly disagree (1), disagree (2), undecided (3), agree (4), and strongly agree (5) [17]. The online questionnaire also included open-ended questions about the advantages and limitations of the ICT system. The questionnaires were administered using Google Forms, an online survey administration software. Participants could access questionnaires through a URL and were able to complete the survey at any time or place, thereby ensuring privacy and honesty. Only participants who agreed to the instructions were invited to complete the survey. Results were processed using SPSS Statistics, version 22.0 (IBM Corp). Questionnaire items were analyzed using frequencies, percentages, means, and standard deviations.

### Focus Group Interviews

Focus group interviews were used for qualitative data collection. One of the distinct features of this method is group dynamics; hence, the type and range of data generated through the social interactions of the group are often deeper and richer than those obtained from one-on-one interviews [18]. The optimum size of focus groups is 6 to 8 participants, excluding researchers, but focus groups can be successful with as few as 3 and as many as 14 participants [19].

Three members of the research team (YSB, MP, and JSL) facilitated the focus groups. The facilitators all have a background in medical informatics and were experienced in conducting focus groups.

In this study, the participants of the focus group interviews were health care professionals (ie, physicians and nurses) who had experience using ICT-based patient management systems in the LTSC of SNUH. The participants were divided into two groups—physicians (n=5) and nurses (n=5)—and attended two sets of interviews in April 2020.

All participating health care professionals provided consent to participate, and they were presented with structured open-ended questions regarding their needs and possible issues when instituting the ICT-based patient management system in the COVID-19 LTSC of SNUH. Each focus group interview was 60 to 90 minutes in length and ended once the conversation no longer yielded new ideas or opinions (ie, saturation of themes). Each focus group interview was recorded in its entirety, with the researchers writing additional memos when necessary. In order to eliminate bias and improve the reliability and validity of the results, two researchers who participated in a course on qualitative research conducted the interviews. All data coders and analysts were trained in qualitative research. All interviews were recorded with the consent of the participants, and the recordings were transcribed as soon as the interviews were completed. In cases where the transcribed data were not comprehensible or interpretable, another follow-up interview was conducted to enhance the reliability of the data and analysis.

The main open-ended questions were as follows:

1. What do you think should be included in ICT-based patient management systems in LTSCs?
2. What are the limitations in introducing ICT-based patient management systems in LTSCs?
3. What is the expected effect of applying ICT-based patient management systems in LTSCs?
4. What is the most important thing (function, role) of ICT-based patient management systems in LTSCs?
5. What are the anticipated administrative issues when using ICT-based patient management systems in LTSCs?
6. What are the expected clinical problems when using ICT-based patient management systems in LTSCs?
7. Do you think ICT-based patient management systems in LTSCs will improve the efficiency of the COVID-19 treatment process? Specifically, what do you think will help?
8. Do you think the experience of introducing ICT-based patient management systems in LTSCs to future long-term care or home care will be helpful? Specifically, what do you think will help?

Qualitative content analysis was as follows. First, we tried to form an overall opinion by reading the interview contents repeatedly. Second, we carefully read each paragraph and formulated the meaning of each statement. Third, we labeled the codes and categorized them according to the subjects’ experience. Lastly, codes were categorized according to their relationship and connectivity, and the arranged codes were organized according to their hierarchy of importance.

## Results

### Overall ICT-Based System Introduced in the LTSC

#### Mobile App

An Android-based mobile app was developed for the LTSC patients to enable efficient patient management and communication between patients and medical staff. The app consisted of six features: a general guide for patients admitted to the LTSC, a notice board, a symptom questionnaire, vital sign reporting, questions and answers, and push notifications (Multimedia Appendix 1). Patients were instructed to do self-checkups twice a day. Patients received push notifications when they needed to fill out a self-report questionnaire on symptoms and vital signs, when they needed to answer questions, or when medical staff posted new notices on the bulletin board. When the patient filled out a structured questionnaire on the presence or absence of symptoms through the app and input vital signs, the corresponding data were immediately linked to the HIS. Medical staff could also upload general guidance and notices regarding the LTSC or COVID-19.

#### Wearable Device

Patients were asked to use a wearable device to measure vital signs and allow medical staff to monitor them remotely. The VDR-1000 was used for this purpose; this a wearable, medical, multi-signal measurement device that can concurrently measure a patient’s electrocardiogram (ECG), pulse rate, blood pressure (BP), blood oxygen saturation (SpO_2_), respiratory waveform, and respiratory rate. The measured data were transmitted to a central monitoring system (CMS) using Wi-Fi and then forwarded to the SNUH HIS. Medical staff in Mungyeong and Seoul used CMS monitors and the HIS to monitor patient vital signs. CMS software can set alarms with different thresholds for each patient. If a value outside the threshold range was measured, an alarm sounded, allowing medical personnel to respond quickly [5].

### Patient Survey

#### Experiences Using the Wearable, Continuous Vital-Sign Monitoring Device

In total, 12 patients completed the questionnaire regarding the wearable device. Of these, 11 (92%) patients provided general information. The mean age was 25 (SD 6.25) years. Of the 11 patients, 6 (55%) were male and 5 (45%) were female (Table S1 in Multimedia Appendix 2). For usability testing of the wearable device, perceived usefulness had the highest score at 4.45 (SD 0.57) points out of 5, followed by perceived ease of use at 4.30 (SD 0.59) points and satisfaction at 3.98 (SD 0.70) points. Of all wearable device measures, SpO_2_ had the highest satisfaction at 4.03 (SD 0.76) points, followed by ECG at 3.94 (SD 0.92) points and BP at 3.76 (SD 0.96) points (Table 1). Items from the perceived usefulness, ease of use, and satisfaction surveys are shown in Tables 2, 3, and 4, respectively. Of the perceived usefulness items, “data measured through wearable devices can improve the quality of care” and “wearable devices will be useful for medical staff” had the highest scores (mean 4.55, SD 0.52). Of the perceived ease of use items, “it was easy to use the wearable device by looking at the manual” and “the wearable device’s weight is appropriate for use” had the highest scores (mean 4.64, SD 0.50). In contrast, the item “it is convenient to move while wearing the device” had the lowest score (mean 3.55, SD 1.44). Of the satisfaction items, “no discomfort when going to the bathroom” and “no discomfort when moving” had the lowest scores (mean 3.00, SD 1.67, and mean 3.27, SD 1.56, respectively) for the device items as well as for the partial wearable devices.

**Table 1:** Usability testing as measured by perceived usefulness, perceived ease of use, and satisfaction (n=11).

Survey category		Score^a^, mean (SD)
Perceived usefulness		4.45 (0.57)
Perceived ease of use		4.30 (0.59)
**Satisfaction**		
	Wearable device	3.98 (0.70)
	Blood pressure measure	4.03 (0.76)
	Electrocardiogram measure	3.94 (0.92)
	Blood oxygen saturation measure	3.76 (0.96)

^a^Survey items were rated on a 5-point Likert scale, ranging from 1 (strongly disagree) to 5 (strongly agree).

**Table 2:** Perceived usefulness of the wearable, continuous vital-sign monitoring device (n=11).

Survey item	Score^a^, mean (SD)
The wearable device can quickly and easily measure the biomarkers required for medical staff decision making.	4.36 (0.67)
Data measured through the wearable device can improve the quality of care.	4.55 (0.52)
The biomarkers measured with the wearable device can improve the efficiency of treatment.	4.45 (0.69)
The wearable device can improve telemedicine care.	4.36 (0.67)
The wearable device will be useful for medical staff.	4.55 (0.52)

^a^Survey items were rated on a 5-point Likert scale, ranging from 1 (strongly disagree) to 5 (strongly agree).

**Table 3:** Perceived ease of use of the wearable, continuous vital-sign monitoring device (n=11).

Survey item	Score^a^, mean (SD)
It is easy to use the wearable device by referring to the manual.	4.64 (0.50)
The wearable device is designed to be easy to use.	4.18 (1.25)
The wearable device size is appropriate for use.	4.55 (0.52)
The wearable device weight is appropriate for use.	4.64 (0.50)
It is convenient to move while wearing the device.	3.55 (1.44)
The wearable device is convenient to store and manage.	4.27 (0.79)

^a^Survey items were rated on a 5-point Likert scale, ranging from 1 (strongly disagree) to 5 (strongly agree).

**Table 4:** Satisfaction with the wearable, continuous vital-sign monitoring device and its measures (n=11).

Survey item		Score, mean (SD)			
		All measures of the device	Blood pressure measure	ECG^a^ measure	SpO_2_^b^ measure
**Individual item^c^**					
	The shape of the wearable device is adequate	4.18 (0.60)	4.36 (0.81)	4.18 (0.60)	4.27 (0.65)
	The size of the wearable device is adequate	4.36 (0.50)	4.36 (0.81)	4.27 (0.65)	4.27 (0.79)
	The weight of the wearable device is adequate	4.36 (0.50)	4.36 (0.67)	4.27 (0.65)	4.18 (0.75)
	The location of the part implementing the function is appropriate	4.36 (0.50)	4.36 (0.67)	4.18 (0.60)	4.27 (0.79)
	It is convenient to operate	4.36 (0.67)	4.45 (0.69)	4 (1.10)	4.45 (0.69)
	It works stably	4.18 (0.60)	4.27 (0.79)	4.27 (0.65)	4.45 (0.69)
	No discomfort when eating food	4.00 (1.18)	3.18 (1.66)	3.55 (1.51)	3.55 (1.51)
	No discomfort when going to the bathroom	3.00 (1.67)	2.27 (1.85)	3.55 (1.51)	3.27 (1.42)
	No discomfort when moving	3.27 (1.56)	2.45 (1.81)	3.55 (1.51)	3.45 (1.51)
	No discomfort when sleeping	3.73 (1.19)	3.36 (1.69)	3.64 (1.50)	3.55 (1.21)
	No difficulty in connecting the device without assistance	N/A^d^	N/A	4.18 (0.75)	N/A
	When attaching or moving the sensor sticker, there was no strain on the skin	N/A	N/A	3.64 (1.50)	N/A
	No difficulty connecting the SpO_2_ device cup to the finger without assistance	N/A	N/A	N/A	4.45 (0.69)
	Willing to use a home-based wearable device with similar performance in the future	3.91 (1.14)	3.91 (1.22)	3.91 (1.22)	4.18 (0.75)
**All items^e^**					
	Expected score for the wearable device before using it	76.36 (22.92)	85.45 (13.68)	76.00 (21.58)	85.27 (11.39)
	Evaluation score for the wearable device after actually using it	89.36 (9.67)	90.55 (10.55)	85.00 (20.12)	91.36 (7.78)

^a^ECG: electrocardiogram.

^b^SpO_2_: blood oxygen saturation.

^c^Survey items were rated on a 5-point Likert scale, ranging from 1 (strongly disagree) to 5 (strongly agree).

^d^N/A: not applicable; this survey item did not pertain to either the device itself or to the indicated measure.

^e^Participants responded using a scale where the maximum score was 100 points.

#### Experience Using the Mobile App

In total, 12 patients completed the questionnaire regarding the mobile app. The mean age was 27.75 (SD 10.24) years, and 5 out of 12 (42%) patients were female.

In terms of usability of the mobile app, perceived usefulness had the highest mean score of 4.62 (SD 0.48) points out of 5, followed by satisfaction with a mean score of 4.08 (SD 1.41) points and perceived ease of use with a mean of 3.81 (SD 0.41) points (Table 5). All of the perceived usefulness items for the app had a higher score than the wearable device itself, with the item “the self-reporting mobile app will be useful for medical staff” scoring the highest (mean 4.75, SD 0.62) (Table 6). Of the perceived ease of use items, “checking cumulative BP, pulse, or body temperature history information,” “checking push messages from medical staff,” “checking for responses from medical staff,” and “searching my notification history” had the highest scores (combined mean 3.92, SD 0.50). Of the satisfaction items, “installing the mobile app,” “log-in,” and “entering measurement results such as BP, pulse, or body temperature” showed the highest scores (mean 4.50, SD 1.17). The item “entering measurement results such as BP, pulse, or body temperature” scored relatively highly, not only in terms of ease of use but also in satisfaction. However, for perceived ease of use items, “installing the mobile app,” “searching for notice information,” and “inquiring and submitting questionnaire data” received the lowest scores (mean 3.67, SD 0.65; mean 3.67, SD 0.49; and mean 3.67, SD 0.49, respectively). The item “searching my notification history” received the lowest score (mean 3.58, SD 2.02) in satisfaction (Table 7).

**Table 5:** Perceived usefulness, perceived ease of use, and satisfaction with the mobile app (n=12).

Category	Score^a^, mean (SD)
Perceived usefulness	4.62 (0.48)
Satisfaction	4.08 (1.41)
Perceived ease of use	3.81 (0.41)

^a^Survey items were rated on a 5-point Likert scale, ranging from 1 (strongly disagree) to 5 (strongly agree).

**Table 6:** Perceived usefulness of the mobile app (n=12).

Survey item	Score^a^, mean (SD)
The self-reporting mobile app can quickly and easily measure the biomarkers required for medical staff decision making.	4.50 (0.67)
Data measured through the self-reporting mobile app can improve the quality of care.	4.50 (0.67)
The biomarkers measured through the self-reporting mobile app can improve the efficiency of treatment.	4.67 (0.65)
The self-reporting mobile app can improve telemedicine care.	4.67 (0.49)
The self-reporting mobile app will be useful for medical staff.	4.75 (0.62)

^a^Survey items were rated on a 5-point Likert scale, ranging from 1 (strongly disagree) to 5 (strongly agree).

**Table 7:** Perceived ease of use of, and satisfaction with, the mobile app (n=12).

Survey item	Ease of use score^a^, mean (SD)	Satisfaction score^a^, mean (SD)
Installing the mobile app	3.67 (0.65)	4.50 (1.17)
Log-in	3.75 (0.45)	4.50 (1.17)
Getting information about care center guidelines	3.83 (0.58)	3.67 (2.06)
Searching for notice information	3.67 (0.49)	3.92 (1.73)
Inquiring and submitting questionnaire data	3.67 (0.49)	4.42 (1.16)
Entering measurements such as BP^b^, pulse, or body temperature	3.83 (0.39)	4.50 (1.17)
Checking cumulative BP, pulse, or body temperature history information	3.92 (0.51)	4.17 (1.75)
Checking push messages from medical staff	3.92 (0.67)	3.75 (2.09)
Checking for responses from medical staff	3.92 (0.67)	3.75 (2.09)
Searching my notification history	3.92 (0.67)	3.58 (2.02)

^a^Survey items were rated on a 5-point Likert scale, ranging from 1 (strongly disagree) to 5 (strongly agree).

^b^BP: blood pressure.

### Survey of Medical Staff

#### Overview

The medical staff who replied to the questionnaire had average of 13.08 (SD 5.33) years of work experience, and their average age was 37.38 (SD 6.27) years. Among them, 83% (20/24) were nurses and approximately 96% (23/24) were female (Table S2 in Multimedia Appendix 2).

#### Medical Staff Satisfaction With the Mobile App and Web Monitoring System

Among satisfaction items for the mobile app, “self-reporting” had the highest mean score at 4.42 (SD 0.58) points out of 5, followed by “center guidelines” at a mean of 4.29 (SD 0.62) points, “vital sign check” at a mean of 4.21 (SD 0.72) points, “notice information” at a mean of 3.96 (SD 0.62) points, “medical inquiries” at a mean of 3.88 (SD 0.68) points, and “push notifications” at a mean of 3.83 (SD 0.64) points (Table 8).

With regard to satisfaction with using the web monitoring system, only 15 staff out of 24 (63%) had full or partial experience using the system. Among the features, “notice information” showed the highest mean score at 4.20 (SD 0.68) points out of 5, followed by “center guidelines” at a mean of 4.13 (SD 0.72) points, “patient management” at a mean of 4.13 (SD 0.0.64) points, “medical inquires” at a mean of 4.08 (SD 0.67) points, and “message management” at a mean of 3.85 (SD 0.80) points (Table 9).

The total mean perceived usefulness score for the wearable devices when providing medical care was 82.79 (SD 2.77) points out of 100. Of the perceived usefulness items, “the wearable devices can improve telemedicine care” had the highest score (mean 4.33, SD 0.70), while “data measured through the wearable devices can improve the quality of care” and “I’m willing to use wearable devices for providing medical care” had the lowest score (mean 4.13, SD 0.68) (Table 10).

**Table 8:** Medical staff satisfaction with the mobile app (N=24).

Survey item	Score^a^, mean (SD)
Self-reporting	4.42 (0.58)
Center guidelines	4.29 (0.62)
Vital sign check	4.21 (0.72)
Notice information	3.96 (0.62)
Medical inquiries	3.88 (0.68)
Push notifications	3.83 (0.64)

^a^Survey items were rated on a 5-point Likert scale, ranging from 1 (strongly disagree) to 5 (strongly agree).

**Table 9:** Medical staff satisfaction with the web monitoring system (n=19).

Survey item	Score^a^, mean (SD)
Notice information	4.20 (0.68)
Center guidelines	4.13 (0.72)
Patient management	4.13 (0.64)
Medical inquiries	4.08 (0.67)
Message management	3.85 (0.80)

^a^Survey items were rated on a 5-point Likert scale, ranging from 1 (strongly disagree) to 5 (strongly agree).

**Table 10:** Perceived usefulness for the wearable continuous vital sign monitoring device among medical staff (n=19).

Survey item			Score, mean (SD)
**Individual items^a^**			
	The wearable device can quickly and easily measure the biomarkers required for medical staff decision making.	4.17 (0.64)	
	Data measured through the wearable device can improve the quality of care.	4.13 (0.61)	
	The biomarkers measured through the wearable device can improve the efficiency of treatment.	4.17 (0.70)	
	The wearable device can improve telemedicine care.	4.38 (0.65)	
	The wearable device will be useful for medical staff.	4.33 (0.70)	
	I am willing to use the wearable device for providing medical care.	4.13 (0.68)	
Total perceived usefulness for the wearable device when providing medical care^b^			82.79 (12.77)

^a^Survey items were rated on a 5-point Likert scale, ranging from 1 (strongly disagree) to 5 (strongly agree).

^b^Participants responded using a scale where the maximum score was 100 points.

Among the 24 medical staff members who replied to the survey, 23 (96%) answered that the “connection with HIS, such as clinical observation record” was the most crucial function that a wearable device can perform. A total of 20 staff members out of 24 (83%) responded that “accuracy” was important for wearable devices, followed by “ease of use” (16/24, 67%). However, only 5 staff members out of 24 (21%) replied that “variety of measurement data types” was a critical feature. The open-ended responses regarding the requirements for wearable device function are listed below (Textbox 1).

**The most crucial functions that a wearable device must perform according to the medical staff members.**

The most crucial functions that a wearable device must perform:

- I think device accuracy is the most important
- It seems to be possible only if the device is accurate and the patient can use it easily
- Device accuracy, HIS (hospital information system) linkage
- Wireless. Patients are too fragmented because of the cable
- Alarm function when it is not attached properly
- HIS linkage is essential for quick response
- Alarm on error
- The accuracy of the device should be high
- HIS linkage for clinical observation recording and verification
- Accuracy, usability, stability, convenience, and privacy
- Accurate measurement and HIS linkage
- Accuracy should be the top priority
- Function for detecting body temperature
- Linkage with all electronic health records that contain patient vital signs
- Function for detecting blood pressure and pulse rate
- Capability of acquiring specific vital signs personalized to each patient
- Vital sign linkage function
- Automatically analyzes the ECG (electrocardiogram) rhythm based on vital signs and alerts medical staff
- HIS linkage and alarm function
- Personal health records
- Function for detecting vital signs

### Focus Group Interviews of Health Care Professionals

#### Overview

Thematic analysis of the focus group interviews with 5 physicians and 4 nurses yielded three themes: (1) major function requirements, (2) expected outcomes, and (3) potential issues. The themes are summarized and described in Table 11.

**Table 11:** Needs of health care professionals and possible issues with information and communications technology–based management systems for patients with COVID-19.

Themes	Subthemes
Major function requirements	Hospital information system interface Features of the wearable device Additional measurement functions Alarms Features of the mobile app Presenting a reference range Messenger
Expected outcomes	Improvement of patient safety Contribution to reducing the burden on medical staff
Potential issues	Stability and reliability of the device Patient education Accountability Cost and reimbursement

#### Major Function Requirements

##### HIS Interface

The majority of participants mentioned that the HIS interface was the most important major function requirement. The system implemented in the LTSC interfaced with the information acquired from wearable devices and apps with the HIS in real time. Most medical professionals emphasized that information obtained from wearable devices or mobile apps should be immediately linked to the HIS to alert staff to changes in patient status while simultaneously reducing staff workload.

It is essential for remote patient monitoring to properly interface and integrate vital sign data generated from a wearable device or patient-generated data collected in the app (eg, symptom self-reporting and vital sign self-measurement data).

For remote patient monitoring, when an abnormal signal is detected by the patient, the clinical information of the patient in HIS must be inquired at any time.

HIS linkage can reduce human errors that may occur during rewriting and reduce the burden on medical staff.

In addition, this is likely to be important not only for infectious diseases such as COVID-19 but also for other chronic diseases and mental health issues.

If the patient self-reports symptoms such as depression or self-measured blood pressure, blood sugar, etc, and these data are linked to the HIS in real time, it will help medical staff identify changes in the patient's condition promptly.

Since not all the data generated by various devices or apps could be linked to the HIS, it is necessary to structure systems so that important surrogate markers for each disease can be linked. Moreover, it is crucial to distinguish whether the data interfaced with the HIS are self-reported or measured by medical professionals.

##### Features of the Wearable Device

###### Additional Measurement Functions

Although patients admitted to the LTSC were afebrile and had no symptoms or only mild rhinorrhea and cough, 2 patients were later admitted to a nearby hospital due to sudden progression of dyspnea and pneumonia. Therefore, many participants suggested that the ability to continuously and concurrently measure body temperature, oxygen saturation, and respiratory rate of isolated patients is a critical value of the wearable device.

In order to treat COVID-19 patients not face-to-face, it is most important to have a device that can measure body temperature and oxygen saturation well.

If the patient self-measures the respiratory rate, it is often a high or low value because the patient has difficulty with self-measuring the respiratory rate.

It would be nice if there was an auscultation function that remotely hears lung sounds when the patient puts the device to the chest. I think that would help us know what is going on with pneumonia.

###### Alarm

When an abnormal value is detected, an alert sound can be used to notify both medical staff and the patient. If the alarm goes off due to wearing the device incorrectly, the patient can reattach the device after checking the manual. If an abnormal value is recorded, medical staff can preemptively respond to the alarm.

By adding an alarm function, the patient can recognize whether it is a false signal, and medical staff can check whether it is an error or an actual abnormality.

##### Features of the Mobile App

###### Presenting a Reference Range

Patients measured vital signs by themselves using the symptom questionnaire and vital sign reporting functions, entered them into the app, and self-reported symptoms. Patients wondered if their vital signs were within normal range. In addition, when a value outside the reference range is measured, medical staff should be able to recognize it at a glance, such as using color indicators.

In the case of vital signs, if they deviate from the reference range, it would be better to display them in a different color.

###### Messenger

Some medical staff emphasized integrating a messenger function into the mobile app. LTSCs were originally public or private facilities that were modified to accommodate and quarantine patients with COVID-19. Therefore, continuous education was required for patients on how to use the facility, how to self-measure vital signs, rules to be observed during quarantine in facilities other than medical institutions, how to dispose of waste, and how to communicate with medical staff when abnormal symptoms occurred.

#### Expected Outcomes

##### Improvement in Patient Safety

Since the data measured by the wearable device are directly linked to the HIS, it reduces the potential for human errors that can occur when manually inputting data into the HIS.

##### Reducing the Burden on Medical Staff

One nurse had to virtually meet about 20 patients at least twice a day. In each consultation, the patient’s vital signs, respiratory symptoms potentially related to COVID-19, digestive symptoms potentially related to COVID-19, and mental health concerns, such as depression and anxiety, were checked. Therefore, each virtual consultation took a considerable amount of time, and the burden on the medical staff was substantial. After the introduction of the electronic medical examination system and the patient mobile app, patients could report their symptoms on their own before starting a scheduled consultation and automatically link them to the HIS so that medical staff could check before starting the virtual consultation.

After the patients could input self-monitoring data into the app and transmit it directly to HIS, the time required was reduced from more than 20 minutes per patient to less than 10 minutes if there were no particular problems.

#### Potential Issues

##### Stability and Reliability of the Device

The stability of the wearable device and the reliability of the measured values are very important. The VDR-1000 used in the LTSC could measure ECG, BP, respiratory rate, heart rate, and SpO_2_ at the same time. For accurate measurement, patients had to connect to wired ECG, BP, and SpO_2_ sensors on the device by themselves while sitting still for 3 to 5 minutes. It is a very sensitive device in which the measured value changes even with small movements.

Because the LTSC had active patients with mild disease, they found it difficult to sit still for five minutes and measure vital signs.

##### Patient Education

Training on management and education of wearable devices or apps is required, as patient familiarity with information technology (IT) varies. Patients who were unfamiliar with using video calls or mobile apps took a considerable amount of time to get used to non–face-to-face treatment, and the medical staff in charge had to repeatedly educate the patient.

##### Accountability

Most medical practitioners agreed that the responsibilities of telemedicine, including diagnosis and prescription, should be established. Remote medical treatments provided by LTSCs were temporarily permitted for COVID-19 outbreaks in certain areas, but the scope of responsibility of medical personnel who perform remote medical treatments should be clarified in preparation for the post–COVID-19 era.

##### Cost and Reimbursement

It is necessary to set an appropriate price for wearable devices that the patient will use. Even if a patient has the opportunity to use a wearable device as part of a non–face-to-face treatment, if it is too expensive, the actual patient’s needs will not be met. In addition, if an appropriate fee for telemedicine is not established, the use of various remote medical solutions and wearable devices capable of remote monitoring will be limited.

## Discussion

### Principal Findings

We administered a questionnaire to clinically healthy patients with COVID-19 and medical staff that included items measuring perceived usefulness, ease of use, and satisfaction with the ICT-based system introduced by SNUH in an LTSC. In addition, focus group interviews were conducted with medical staff to obtain qualitative insights in order to seek future development directions.

Since the COVID-19 pandemic, telemedicine has been spotlighted as a useful way to respond to infectious diseases [20]. Originally, telemedicine was introduced for medically underprivileged areas. With the development of technology over recent decades, various wearable devices, sensors, and platforms have gradually expanded the application of telemedicine to noncommunicable diseases [21,22], infectious diseases, and psychiatric diseases [23]. Clinical consultations through video calls are associated with high patient satisfaction [21,24], and there are no differences in clinical outcomes [22,23,25] compared to face-to-face treatment [26]. However, this is the first time that telemedicine has been performed during a global catastrophe like COVID-19, and research evaluating objective effects is sparse. One study investigated patient satisfaction with telemedicine during the COVID-19 pandemic [27]. However, there have been few studies evaluating the usefulness or satisfaction of both patients and medical professionals. Strengths of our study include evaluation of patient satisfaction as well as evaluation of medical staff satisfaction and conducting of focus group interviews. We found that patients expected that the use of a wearable device would improve quality of care and be helpful in medical treatment. Most respondents reported no major problems with the use of the wearable device, but they complained of discomfort when moving while wearing the device. The wearable device we adopted has multiple lines to accurately measure several vital signs at the same time, which may cause inconvenience to users who are relatively healthy, like those admitted to the LTSC. Therefore, in the future, it is crucial to consider introducing a simple device, such as a wrist monitor, for convenient remote measurement of vital signs in clinically healthy patients with COVID-19. However, the VDR-1000 allowed medical staff to monitor patients’ vital signs at a glance through the CMS, even from the Seoul central monitoring center. In addition, it is possible to selectively search and view past data, or to set an alarm that would sound when the device measures a value outside a specific range for each patient, helping with medical treatment. Above all, the measured data were directly linked to the vital sign sheet in the SNUH HIS, which facilitated medical treatment and reduced potential human errors that may occur during the normal process of recording, transcription, and data entry. In the mobile app satisfaction survey, “push notification” had the lowest score among both patients and medical staff. This notification function is the most recently implemented in the mobile app and was developed to provide advanced notice to patients to self-report symptoms and vital signs at the appointed times twice a day. Unfortunately, at the introduction of the mobile app, only the initial function of sending a message or push alarm to all patients in the LTSC was implemented, and it was not possible to give specific alarms to patients assigned to each medical staff member. The focus group interview results showed the need for a messenger function, including SMS and an alarm function, to deliver and communicate patient-specific messages. Development of such functions should be integrated into a future non–face-to-face care platform for management of patients with COVID-19.

The expected outcomes obtained through focus group interviews regarding the ICT-based system introduced in the LTSC of SNUH could contribute to improving patient safety and reducing the burden on medical staff. As a potential issue, there was an opinion that the device used for remote patient monitoring should be stable and reliable. In addition, because each patient has a different degree of familiarity with IT, different amounts of education will be required. In fact, the United States Health Insurance Portability and Accountability Act (HIPAA) has allowed the use of commonly used video call systems, such as FaceTime, Google Hangouts, and Skype, for video consultation [26]. In addition, a new type of position, such as a technological liaison or coordinator, may be required to overcome the hurdle of patient unfamiliarity with telemedicine [28,29].

### Telemedicine in Korea

Korea is one of the few countries where telemedicine is completely banned. The government, the medical community, civic groups, and politicians have different opinions. In particular, there is an opinion that Korea should take a different approach from those in countries where telemedicine is active, due to the characteristics of Korea’s medical system, which provides easy medical access with low medical costs. However, one of the best ways in which various digital technologies, such as communication, sensors, the cloud, and information security, can be integrated in the medical field is through the application of telemedicine. In Korea, even if an implantable defibrillator with a remote monitoring function is implanted in patients with arrhythmias, the function is turned off due to policy regulations. Due to these sanctions, there has been little discussion about payment structures related to telemedicine, medical information systems, cost and reimbursement, and security issues. During the recent COVID-19 pandemic, studies have suggested that active use of telemedicine technologies for triage, monitoring, communication, and critical care management [30] may be useful in Korea. It is necessary to selectively allow telemedicine in areas where there is a medical need, and to systematically establish and operate a related technical base. In Korea, at the end of February 2020, phone consultation was temporarily allowed to ensure access to medical care in the COVID-19 situation. By October 25, 2020, up to 950,000 cases of non–face-to-face treatment have been implemented [31]. The Korean government is actively promoting telemedicine. By 2025, 18 smart hospitals using ICT are planned to be built; by 2021, imaging equipment will be provided to 5000 clinic-level medical institutions. The government plans to increase the number of clinics by 500 in 2021, to create a total of 1000 clinics. The budget for this is US $93 million by the end of 2021 [31]. However, considering the complicated medical system of Korea, telemedicine must be addressed carefully. Since there are many stakeholders related to telemedicine, a process for social consensus is necessary. The government should play a leading role in this process, and consensus through in-depth medical, technical, financial, regulatory, and industrial expert discussions considering the complexity and specificity of this issue will be needed. Current evolving IT could be very useful for collecting meaningful data on large cohorts as well as infectious diseases [32]. It can be used not only in relation to COVID-19 but also throughout the patient chain of care, such as during prehospital [33], inpatient [34], and postdischarge stages [35,36]. In addition, selective consideration may be needed to adopt telemedicine in health care. Infectious diseases as well as chronic diseases, mental illness, postoperative patient management, and home care fall into a “gray zone” of existing medical care where telemedicine is particularly useful. In order to collect patient-derived data produced by various devices, sensors, and platforms, a vendor-neutral platform is needed, and two-way communication between medical staff and patients is possible only when data are integrated with a standardized protocol to be linked to the HIS [37,38]. Moreover, it is necessary to evaluate the evidence-based effectiveness of telemedicine and compare it to existing face-to-face treatment: whether patient outcomes of telemedicine are similar or improved, whether the overall quality of medical care is improved, and whether it helps to improve medical productivity and costs. It must be proven to be effective.

### Strengths and Limitations

The most powerful strength of this study is evaluation of the perspectives of both patients and medical staff who had participated in newly developed COVID-19–specific non–face-to-face consultation solutions. Our findings will be helpful when setting up telemedicine for contagious diseases as well as noncommunicable diseases. One limitation is that there were insufficient COVID-19–specific investigations. While applying telemedicine to COVID-19, it is important to understand equipment, legislative considerations, coding, logistic concerns, quality of care, cost-effectiveness, and clinical outcomes. In addition, we used a cross-sectional design with which we could not evaluate long-term outcomes due to the short 36-day operation period of the LTSC. In this context, further studies are needed to evaluate telemedicine specific to COVID-19 to improve overall clinical care and health management.

### Conclusions

We demonstrated patient and medical professional satisfaction with, and usability of, an ICT-based system for clinically healthy patients with COVID-19. Our findings support the usefulness of telemedicine and wearable devices during a global infectious crisis.

## Appendix

Multimedia Appendix 1 Screenshots of the mobile app in Korean (left) and English (right) showing the six features.

Multimedia Appendix 2 Supplementary tables.

## Abbreviations

BP: blood pressure
CMS: central monitoring system
ECG: electrocardiogram
HIPAA: Health Insurance Portability and Accountability Act
HIS: hospital information system
ICT: information and communications technology
IT: information technology
LTSC: living and treatment support center
SNUH: Seoul National University Hospital
SpO_2_: blood oxygen saturation
VDR: vital-sign data recorder
WHO: World Health Organization

## References

[1] (2020). Coronavirus disease (COVID-19): Weekly epidemiological update and weekly operational update. World Health Organization.

[2] (2020). WHO Coronavirus (COVID-19) Dashboard. World Health Organization.

[3] (2020). Current Status of Response to COVID-19 and Future Plans.

[4] Coronavirus Disease-19, Republic of Korea: Weekly updates for countries with major outbreaks. Ministry of Health and Welfare.

[5] Bae YS, Kim KH, Choi SW, Ko T, Jeong CW, Cho B, Kim MS, Kang E (2020). Information technology-based management of clinically healthy COVID-19 patients: Lessons from a living and treatment support center operated by Seoul National University Hospital. J Med Internet Res.

[6] Jin C, Chen W, Cao Y, Xu Z, Tan Z, Zhang X, Deng L, Zheng C, Zhou J, Shi H, Feng J (2020). Development and evaluation of an artificial intelligence system for COVID-19 diagnosis. Nat Commun.

[7] Seshadri DR, Davies EV, Harlow ER, Hsu JJ, Knighton SC, Walker TA, Voos JE, Drummond CK (2020). Wearable sensors for COVID-19: A call to action to harness our digital infrastructure for remote patient monitoring and virtual assessments. Front Digit Health.

[8] Mei X, Lee H, Diao K, Huang M, Lin B, Liu C, Xie Z, Ma Y, Robson PM, Chung M, Bernheim A, Mani V, Calcagno C, Li K, Li S, Shan H, Lv J, Zhao T, Xia J, Long Q, Steinberger S, Jacobi A, Deyer T, Luksza M, Liu F, Little BP, Fayad ZA, Yang Y (2020). Artificial intelligence-enabled rapid diagnosis of patients with COVID-19. Nat Med.

[9] Wong CK, Ho DTY, Tam AR, Zhou M, Lau YM, Tang MOY, Tong RCF, Rajput KS, Chen G, Chan SC, Siu CW, Hung IFN (2020). Artificial intelligence mobile health platform for early detection of COVID-19 in quarantine subjects using a wearable biosensor: Protocol for a randomised controlled trial. BMJ Open.

[10] Tsikala Vafea M, Atalla E, Georgakas J, Shehadeh F, Mylona EK, Kalligeros M, Mylonakis E (2020). Emerging technologies for use in the study, diagnosis, and treatment of patients with COVID-19. Cell Mol Bioeng.

[11] Vaishya R, Javaid M, Khan IH, Haleem A (2020). Artificial intelligence (AI) applications for COVID-19 pandemic. Diabetes Metab Syndr.

[12] Singh RP, Javaid M, Haleem A, Suman R (2020). Internet of things (IoT) applications to fight against COVID-19 pandemic. Diabetes Metab Syndr.

[13] Nasajpour M, Pouriyeh S, Parizi RM, Dorodchi M, Valero M, Arabnia HR (2020). Internet of things for current COVID-19 and future pandemics: An exploratory study. J Healthc Inform Res.

[14] Davis FD (1989). Perceived usefulness, perceived ease of use, and user acceptance of information technology. MIS Q.

[15] Rahimi B, Nadri H, Lotfnezhad Afshar H, Timpka T (2018). A systematic review of the technology acceptance model in health informatics. Appl Clin Inform.

[16] Venkatesh V, Morris MG, Davis GB, Davis FD (2003). User acceptance of information technology: Toward a unified view. MIS Q.

[17] McLeod S (2019). Likert scale definition, examples and analysis. Simply Psychology.

[18] Kitzinger J (1994). The methodology of focus groups: The importance of interaction between research participants. Sociol Health Illn.

[19] Gill P, Stewart K, Treasure E, Chadwick B (2008). Methods of data collection in qualitative research: Interviews and focus groups. Br Dent J.

[20] Hollander JE, Carr BG (2020). Virtually perfect? Telemedicine for Covid-19. N Engl J Med.

[21] Dorsey ER, Venkataraman V, Grana MJ, Bull MT, George BP, Boyd CM, Beck CA, Rajan B, Seidmann A, Biglan KM (2013). Randomized controlled clinical trial of "virtual house calls" for Parkinson disease. JAMA Neurol.

[22] Su D, Zhou J, Kelley MS, Michaud TL, Siahpush M, Kim J, Wilson F, Stimpson JP, Pagán JA (2016). Does telemedicine improve treatment outcomes for diabetes? A meta-analysis of results from 55 randomized controlled trials. Diabetes Res Clin Pract.

[23] Fortney JC, Pyne JM, Kimbrell TA, Hudson TJ, Robinson DE, Schneider R, Moore WM, Custer PJ, Grubbs KM, Schnurr PP (2015). Telemedicine-based collaborative care for posttraumatic stress disorder: A randomized clinical trial. JAMA Psychiatry.

[24] Sabesan S, Simcox K, Marr I (2012). Medical oncology clinics through videoconferencing: An acceptable telehealth model for rural patients and health workers. Intern Med J.

[25] de Jong MJ, van der Meulen-de Jong AE, Romberg-Camps MJ, Becx MC, Maljaars JP, Cilissen M, van Bodegraven AA, Mahmmod N, Markus T, Hameeteman WM, Dijkstra G, Masclee AA, Boonen A, Winkens B, van Tubergen A, Jonkers DM, Pierik MJ (2017). Telemedicine for management of inflammatory bowel disease (myIBDcoach): A pragmatic, multicentre, randomised controlled trial. Lancet.

[26] Calton B, Abedini N, Fratkin M (2020). Telemedicine in the time of coronavirus. J Pain Symptom Manage.

[27] Ramaswamy A, Yu M, Drangsholt S, Ng E, Culligan PJ, Schlegel PN, Hu JC (2020). Patient satisfaction with telemedicine during the COVID-19 pandemic: Retrospective cohort study. J Med Internet Res.

[28] Vidal-Alaball J, Acosta-Roja R, Pastor Hernández N, Sanchez Luque U, Morrison D, Narejos Pérez S, Perez-Llano J, Salvador Vèrges A, López Seguí F (2020). Telemedicine in the face of the COVID-19 pandemic. Aten Primaria.

[29] Greenhalgh T, Koh GCH, Car J (2020). Covid-19: A remote assessment in primary care. BMJ.

[30] Hong Z, Li N, Li D, Li J, Li B, Xiong W, Lu L, Li W, Zhou D (2020). Telemedicine during the COVID-19 pandemic: Experiences from Western China. J Med Internet Res.

[31] 950,000 temporary phone prescriptions this year. MEDI:GATE NEWS.

[32] Fagherazzi G, Goetzinger C, Rashid MA, Aguayo GA, Huiart L (2020). Digital health strategies to fight COVID-19 worldwide: Challenges, recommendations, and a call for papers. J Med Internet Res.

[33] Chou E, Hsieh Y, Wolfshohl J, Green F, Bhakta T (2020). Onsite telemedicine strategy for coronavirus (COVID-19) screening to limit exposure in ED. Emerg Med J.

[34] Zhai Y, Wang Y, Zhang M, Hoffer Gittell J, Jiang S, Chen B, Cui F, He X, Zhao J, Wang X From isolation to coordination: How can telemedicine help combat the COVID-19 outbreak?. medRixv..

[35] Singh RP, Javaid M, Haleem A, Suman R (2020). Internet of things (IoT) applications to fight against COVID-19 pandemic. Diabetes Metab Syndr.

[36] Nasajpour M, Pouriyeh S, Parizi RM, Dorodchi M, Valero M, Arabnia HR (2020). Internet of things for current COVID-19 and future pandemics: An exploratory study. J Healthc Inform Res.

[37] Jamil F, Ahmad S, Iqbal N, Kim D (2020). Towards a remote monitoring of patient vital signs based on IoT-based blockchain integrity management platforms in smart hospitals. Sensors (Basel).

[38] Soltan AAS, Kouchaki S, Zhu T, Kiyasseh D, Taylor T, Hussain ZB, Peto T, Brent AJ, Eyre DW, Clifton DA (2021). Rapid triage for COVID-19 using routine clinical data for patients attending hospital: Development and prospective validation of an artificial intelligence screening test. Lancet Digit Health.

