# Supplementary figures and images for: Satisfaction and Usability of an Information and Communications Technology–Based System by Clinically Healthy Patients With COVID-19 and Medical Professionals: Cross-sectional Survey and Focus Group Interview Study
Source: JMIR Form Res. 2021 Aug 26;5(8):e26227. doi: 10.2196/26227 (PMC8396536; doi:10.2196/26227)

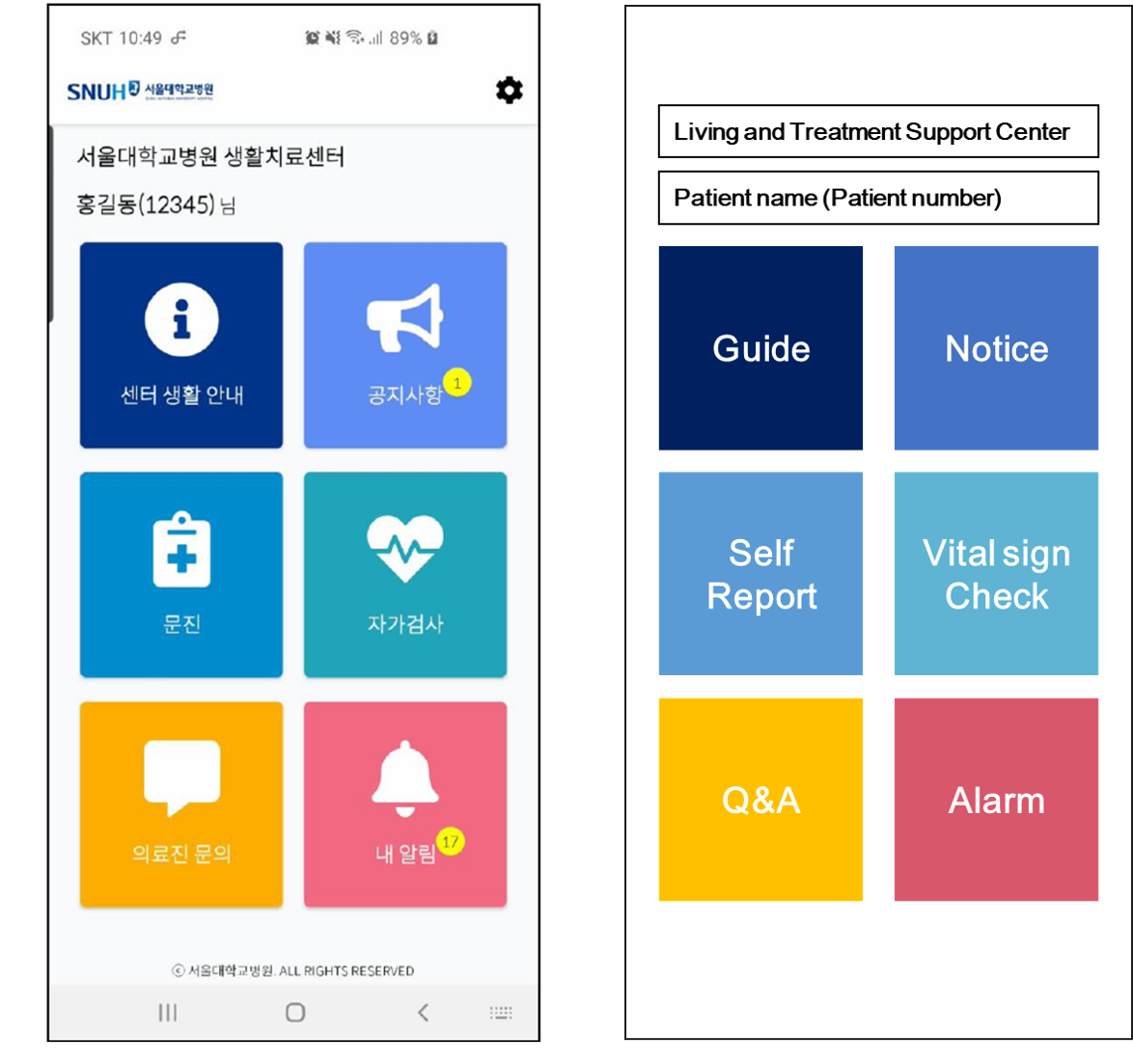

Supplement: Multimedia Appendix 1 [file formative_v5i8e26227_app1.png]
