# Supplementary material for: Satisfaction and Usability of an Information and Communications Technology–Based System by Clinically Healthy Patients With COVID-19 and Medical Professionals: Cross-sectional Survey and Focus Group Interview Study
Source: JMIR Form Res. 2021 Aug 26;5(8):e26227. doi: 10.2196/26227 (PMC8396536; doi:10.2196/26227)
Supplement: Multimedia Appendix 2 [file formative_v5i8e26227_app2.docx]

**Table S1. General characteristics of patients who responded to the questionnaire**

| **Categories** | | **N** | **%** |
| --- | --- | --- | --- |
| Gender | Male | 6 | 54.5 |
|  | Female | 5 | 45.5 |
| Occupation | Specialist | 1 | 9.1 |
|  | office worker | 1 | 9.1 |
|  | Students | 7 | 63.6 |
|  | Inoccupation | 2 | 18.2 |
| Education | High school | 7 | 63.6 |
|  | College | 4 | 36.4 |
| Residence | Single residence | 1 | 9.1 |
|  | Living with family | 9 | 81.8 |
|  | Living with relatives | 1 | 9.1 |
| How to get health information | Smartphone | 6 | 54.5 |
|  | Smartphone & Computer | 1 | 9.1 |
|  | Smartphone & TV | 2 | 18.2 |
|  | Smartphone & Computer & TV | 2 | 18.2 |
| Frequency for searching health information | Every day | 1 | 9.1 |
|  | 1~2 times per week | 3 | 27.3 |
|  | 2~3 times per month | 3 | 27.3 |
|  | once a month | 2 | 18.2 |
|  | once per 3~4 month | 2 | 18.2 |
| Age | Mean ± SD | 25 ± 6.25 | |

**Table S2. General characteristics of medical staffs who responded to the questionnaire**

|  | | Min | | Max | Mean | | SD |
| --- | --- | --- | --- | --- | --- | --- | --- |
| Age | | 28.00 | | 54.00 | 37.38 | | 6.27 |
| Working experiences | | 6.00 | | 30.00 | 13.08 | | 5.33 |
|  | | | N | | | % | |
| Occupation | Nurse | | 20 | | | 83.3 | |
|  | Doctor | | 4 | | | 16.7 | |
| Gender | Female | | 23 | | | 95.8 | |
|  | Male | | 1 | | | 4.2 | |
